# “Neutral Satisfied” Patients Should Not Be Dichotomized to “Satisfied” or “Dissatisfied” in Patient-Reported Outcomes After Total Knee Arthroplasty †

**DOI:** 10.3390/jcm14134482

**Published:** 2025-06-24

**Authors:** Jason M. Cholewa, Mike B. Anderson, Krishna R. Tripuraneni, Jess H. Lonner, Roberta E. Redfern

**Affiliations:** 1 Zimmer Biomet, 345 E Main St, Warsaw, IN 46580, USA; mike.anderson@zimmerbiomet.com (M.B.A.); roberta.redfern@zimmerbiomet.com (R.E.R.); 2 New Mexico Orthopaedic Associates, 2100 Louisiana Blvd NE, Suite 410, Albuquerque, NM 87110, USA; tripuranenikr@nmortho.net; 3 Rothman Orthopaedic Institute, 925 Chestnut St, Philadelphia, PA 19107, USA; jess.lonner@rothmanortho.com

**Keywords:** patient-reported outcome measures, knee society score, arthroplasty registries, satisfaction

## Abstract

**Background:** The purpose of this study was to clinically characterize neutrally satisfied patients and compare outcomes between satisfied, dissatisfied, and neutral patients. **Methods:** This was a secondary analysis from data collected in a multicenter longitudinal cohort study comprising total knee arthroplasty (TKA) patients using a digital care management platform. The Knee Society Score (KSS) satisfaction survey was administered at post-operative 90 days, and dissatisfaction was defined as a composite score of less than 20, satisfied as a score equal to or greater than 30, and neutral as a score of 20 up to 29. Patient-reported outcome measures (PROMs) were assessed pre-operatively and at post-operative one, three, six, and twelve months. **Results:** Approximately 58% of patients were satisfied (n = 1486), 29.4% neutral (n = 747), and 12.2% dissatisfied (n = 311). Neutral and dissatisfied patients were younger and more likely to be female and had lower pre-operative KSS scores compared to satisfied patients, though statistical differences were found between all groups. Pre-operative pain was significantly less in satisfied compared to neutral or dissatisfied patients. Changes in the pre-operative Knee Injury and Osteoarthritis Outcome Score for Joint Replacement (KOOS JR) displayed significant differences between all groups at all time points, with greater improvements in satisfied versus neutral patients and neutral versus dissatisfied patients. Similarly, satisfied patients experienced significantly greater improvements in pain and KSS scores at post-operative three months, and neutral patients improved more than dissatisfied patients. **Conclusions:** Neutral patients present with distinctively different clinical outcomes compared to satisfied or dissatisfied patients and should be classified separately as neutral.

## 1. Introduction

Total knee arthroplasty (TKA) is one of the most prevalent elective surgeries performed in the US [1,2] and is considered a highly effective treatment in end-stage knee osteoarthritis [3]. In addition to implant survival, TKA success may be measured objectively with range of motion (ROM) and functional outcomes or via subjective measures, like pain, patient-reported outcome measures (PROMS), and satisfaction. Satisfaction is now recognized as an important measure of healthcare [4,5], and reporting may be required by government programs [6].

Despite continued advancements in implants, surgical techniques, and technology, the level of patient dissatisfaction with TKA has continued to be reported to be approximately 15–20% over the past three decades [7,8,9,10,11,12,13]. Studies that assess satisfaction as a primary outcome after TKA have exhibited considerable variability [14,15], with patient dissatisfaction rates reported to be as low as 5% [16] and as high as 25% [17,18,19]. The high variabilities in patient dissatisfaction reported between studies may be attributed in part to the instruments used. In a systematic review of 208 studies, Khalenberg et al. [20] reported that 24.6% used a numeric or visual analog scale (VAS), 10.1% used a binary (yes/no) response, 6.3% assessed satisfaction via willingness to undergo surgery again, and the remaining 61% used an ordinal scale (e.g., very satisfied, satisfied, neutral, dissatisfied, and very dissatisfied). Additionally, satisfaction may be assessed as a single question (i.e., “How satisfied are you with your knee procedure?”) or may be a composite score comprising responses to multiple questions regarding satisfaction with a number of facets of knee health and function.

Heterogeneity within the answers supplied on the ordinal scales used to assess satisfaction also exists, with some authors including a neutral option [9,17,19,21,22,23,24] and others excluding it [25,26,27,28,29]. The International Society of Arthroplasty Registries (ISAR) currently endorses using an ordinal scale, asserts many patients that select neutral do so because they do not want to appear negative, and recommends classifying neutral patients as dissatisfied [30]. Though most studies follow this recommendation [9,19,21,22,23,24,31,32,33,34,35,36], some studies either report neutral as a separate category [21,37,38] or censor/exclude neutral responses in the dichotomization [17,28].

To the best of our knowledge, only one study has reported on the demographic and clinical characteristics of patients that are neither satisfied nor dissatisfied with their TKA. Ulivi et al. [38] reported no difference between neutral and satisfied or dissatisfied patients for demographics or the Short Form (SF-36) Physical and Mental component at post-operative 6 or 12 months. At post-operative five years, neutral patients reported significantly greater Physical Functioning scores of the Knee Injury and Osteoarthritis Outcome Score (KOOS) compared to dissatisfied patients but significantly lower ones than satisfied patients. However, the primary purpose of Ulivi et al. was not to assess differences between neutral and satisfied or dissatisfied patients, and their study was limited by a small sample size at a single institution. Therefore, more data is necessary to determine whether to classify neutral satisfaction patients as dissatisfied or a separate category. The purpose of this study was to clinically characterize neutrally satisfied patients and compare outcomes between satisfied, dissatisfied, and neutral patients.

## 2. Methods

This study is a secondary analysis of data collected during a prospective cohort study (A Prospective Multicenter Longitudinal Cohort Study of the mymobility Platform, clinicaltrials.gov: NCT# 03737149), approved by an Institutional Review Board (WCG IRB # 20182013). The global study has been described in previous reports and consists of three phases, including a pilot phase, a randomized controlled trial (RCT) phase, and a longitudinal cohort phase [39,40,41,42]. Data reviewed in this study was limited to the longitudinal cohort in participants who underwent TKA between November 2018 and October 2023.

Inclusion criteria included the following: individuals at least 18 years of age, who owned and maintained an iPhone (Apple, Cupertino, CA, USA) capable of pairing to the Apple Watch compatible with a mobile app (mymobility^®^ Zimmer Biomet, Warsaw, IN, USA), and were able to ambulate independently with no more than a single cane/single crutch pre-operatively. Potential participants were excluded if they were a current alcohol or drug abuser, as defined by the investigator; had systemic inflammatory arthropathies that would interfere or compromise the activity profiles within the study; were participating in any other surgical intervention, physical therapy, or pain management study which would compromise the results of this study; or required simultaneous or staged bilateral replacements, staged less than 90 days apart. To avoid potential selection bias, each investigator offered study participation to each eligible patient presenting as a candidate for primary unilateral TKA sequentially. Eligible patients who chose to participate in the study provided written informed consent prior to any study-related procedures being performed.

Satisfaction was assessed via the 2011 Knee Society Scoring System (KSS) [43] at post-operative three months. The KSS satisfaction subscale contains five questions about the affected joint related to pain while sitting, pain while lying in a bed, pain while getting out of bed, the ability to perform light household duties, and the ability to perform leisure recreational activities. Each item is answered on a 5-point ordinal scale. For each item, “very satisfied” received 8 points, “satisfied” received 6 points, “neutral” received 4 points, “dissatisfied” received 2 points, and “very dissatisfied” received 0 points to create a KSS composite score of up to 40 points. Patients were grouped according to KSS satisfaction score into satisfied (≥30), neutral (<30 to ≥20), and dissatisfied (<20). These values correspond to an average response to all five questions as satisfied (at least 6 points per item, on average), neutral (between 4 and 5.9 points per item, on average), and dissatisfied (less than 4 points per item, on average), respectively. Other studies have also used ≥30 as a cut-off point to dichotomize satisfied and dissatisfied patients according to the KSS satisfaction score [44,45,46].

The Knee Injury and Osteoarthritis Outcome Score for Joint Replacement (KOOS JR) was evaluated pre-operatively and at post-operative one, three, six, and twelve months. The numeric rating scale (0–10) for pain, active flexion range of motion (ROM), and the KSS satisfaction subscale were assessed pre-operatively and at post-operative one and three months. Additionally, patients were asked to indicate whether they had performed specific activities of daily living (independent walking, independent driving, return to work, sexual activity, light household work, and heavy household work) and the date they first returned to the respective activity at post-operative three months, as previously described [47].

### Data Analyses

Continuous variables are reported as the mean with the standard deviation (SD) and range (minimum–maximum) and were compared between post-operative satisfaction groups by one-way ANOVA with post hoc Tukey pairwise comparisons in the event of a statistically significant overall model. Categorical data are presented as a frequency and percentage and compared by a chi-square test or Fisher’s Exact test, where appropriate. All analyses were performed with SAS Enterprise Guide version 7.1 (2014, SAS Institute, Inc., Cary, NC, USA). A *p*-value < 0.05 was considered statistically significant.

## 3. Results

At post-operative three months, approximately 58% of participants were satisfied (n = 1500), 29% neutral (n = 756), and 12% dissatisfied (n = 314). The three-month KSS satisfaction scores for dissatisfied, neutral, and satisfied participants were 12.8 ± 5.3, 24.6 ± 2.9, and 35.3 ± 3.8, respectively (*p* < 0.001). Pre-operatively, neutral and dissatisfied participants tended to be younger, more often female, and less satisfied with the pain and function related to their native knees and reported higher levels of pain (Table 1). Pairwise comparisons showed significant differences between all three groups with regard to age and pre-operative satisfaction (all *p* < 0.05). Considering pre-operative pain, only those patients who were satisfied after surgery differed from the other groups on pairwise comparison. However, the differences in pre-operative satisfaction and pain scores were negligible and may not be clinically relevant. On average, participants were dissatisfied with their knee pain and function pre-operatively across all three groups, where the average score across all participants was 14.1 ± 7.94 points. There were no differences in Charnley classification or alignment pre-operatively; however, those who were dissatisfied after surgery more often appeared to have joints judged as “tight” by the surgeon prior to intervention.

**Figure 1:**
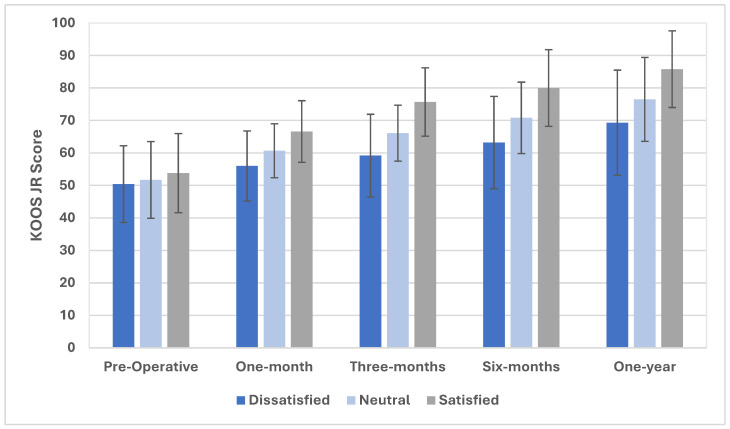
KOOS JR scores for satisfied, neutral, and dissatisfied patients up to post-operative one year.

Pre-operatively, satisfied participants had significantly (*p* < 0.001) higher KOOS JR scores than either neutral or dissatisfied patients, though on pairwise comparison, neutral and dissatisfied patients did not vary significantly from one another on this measure prior to TKA. Significant (*p* < 0.001) differences were found between all groups for the KOOS JR at each time point post-operatively (Figure 1), where patients indicating satisfaction on the KSS subscale instrument demonstrated the highest KOOS JR scores at all intervals. Importantly, the mean differences between the groups continue to diverge up to post-operative six months, surpassing established distribution-based minimal clinically important differences (MCIDs) for the instrument [48]. The change in KOOS JR scores pre-operatively was also significantly different between groups at each follow-up period (Table 2), with a similar trend demonstrating diverging scores between all three groups until post-operative six months, where the difference in change in scores meets MCID criteria between both dissatisfied versus neutral (6.2 points) and neutral and satisfied patients (7.4 points) at one post-operative year and thus may represent clinically significant differences.

**Figure 2:**
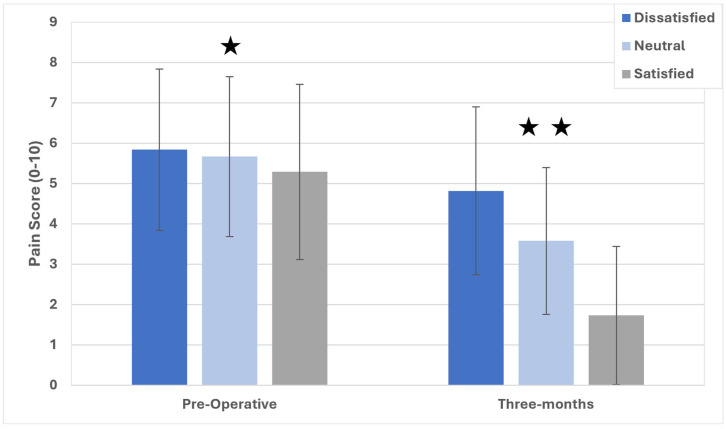
Pain scores for satisfied, neutral, and dissatisfied participants up to post-operative three months. ★ Satisfied participants are significantly different from dissatisfied and neutral ones; ★★ significant differences between all groups.

**Figure 3:**
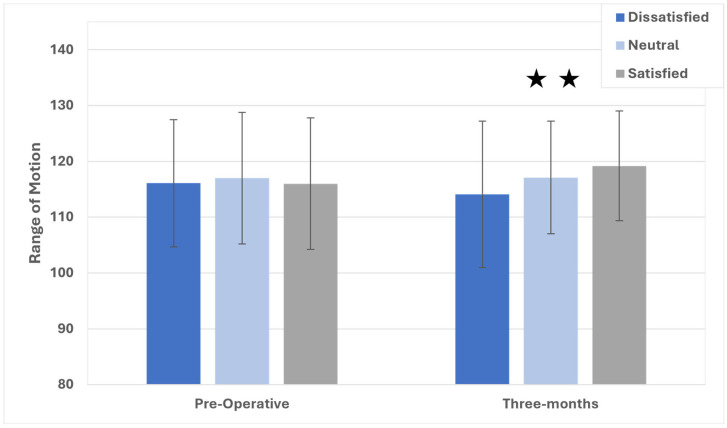
Range of motion scores for satisfied, neutral, and dissatisfied patients up to post-operative three months. ★★ Significant differences between all groups.

There were significant differences (*p* < 0.001) between all groups for changes in pain (Figure 2), where dissatisfied patients reported a 0.99-point reduction pre-operatively, those in the neutral category reported a 2.11-point reduction, and those who were satisfied appreciated a 3.55-point pain reduction from the baseline. At post-operative three months, differences in numeric pain scores met statistical significance between all groups, though only the satisfied versus dissatisfied patients met clinical significance (2-point difference). The results of an investigation of the range of motion (ROM), as measured during in-clinic visits up to post-operative three months, are shown in Figure 3. Pre-operatively, there were no differences in ROM between the groups on ANOVA (*p* = 0.22). At three months following TKA, the overall ANOVA model suggested significant differences in the post-operative range of motion, where satisfied patients exhibited 119.2° ± 9.8°, neutral patients attained 117.1° ± 10.1°, and dissatisfied patients reached 114.1° ± 13.1°. All pairwise comparisons were significant (*p* < 0.05), and all differences were >2°, suggesting clinical significance.

Significant differences were found between all groups (Table 3) in the percentage of participants who returned to walking independently (*p* < 0.001), driving independently (*p* < 0.001), sexual activity (*p* < 0.001), and light (*p* < 0.001) and heavy household activities (*p* < 0.001). A significantly (*p* < 0.001) greater proportion of satisfied participants returned to work than dissatisfied or neutral participants. Additionally, satisfied participants returned to walking independently sooner than neutral or dissatisfied participants (6.5 and 3.4 days, respectively, both *p* < 0.05), driving independently faster than dissatisfied participants (4.4 days, *p* < 0.05), and light household activities faster than either group (7.2 and 2.9 days, both *p* < 0.05). Neutral participants returned to light household activities significantly (4.4 days) sooner than dissatisfied participants (*p* < 0.05). No differences were observed in the time to return to work, sexual activity, or heavy housework in those who attained this goal at post-operative three months.

## 4. Discussion

This prospective cohort study found that participants who indicated neutral satisfaction at post-operative three months presented with unique demographic and clinical outcomes. We found neutral participants to display greater improvements in function, satisfaction, pain, ROM, and certain activities post-operatively compared to dissatisfied participants. However, neutral participants did not achieve the same status in these measures as satisfied participants.

This study was strengthened by its prospective, multicenter, international participant recruitment and large sample size. However, certain limitations need to be addressed before discussing the implications of the findings. In the present study, 58% of patients were satisfied at post-operative three months, which is considerably below the generally accepted rate of 80–85% [7,8,9,10,11,12,13,14]. As a secondary analysis of prospectively collected data for the purpose of evaluating a digital care management platform, we were limited to three-month satisfaction data, whereas the systematic reviews that report 80–85% satisfaction rates only included studies with follow-up periods of at least post-operative one year [8,10,14]. Given that mean satisfaction has been reported to improve from the first three months up to post-operative one year [49], the overall rate of satisfaction in the present study is likely not stagnant. Indeed, the outcomes of our study may differ with longer term follow-ups. However, studies have suggested that differences in satisfaction are apparent as early as post-operative 12 weeks and may be predictive of future outcomes, justifying the use of the metric at this early post-operative interval [50].

Questions about satisfaction with pain relief or specific activities also yield lower rates of satisfaction compared to a general question about overall satisfaction with the outcome [25]. The KSS satisfaction questionnaire used in the present study specifically surveys pain and function and likely contributed to rates of satisfaction lower than those typically reported in the literature. Discrepancies between the rate of satisfaction in the present study and the literature may further be explained by the quantitative definition of satisfaction. In the present study, a KSS score of 30 or greater was considered satisfied, while other studies have defined satisfaction with a KSS score of 20 or greater [51,52]. Nevertheless, the overall mean KSS satisfaction score in the present study of approximately 29 is comparable to other studies that have reported early post-operative KSS satisfaction scores [45,46,49,53]. Our results suggest this tool may be useful in the early post-operative period to distinguish groups whose recovery may be suboptimal. KOOS JR absolute and delta scores were all statistically and clinically different between groups at post-operative six and twelve months [48], suggesting that many of the clinical outcomes found between groups at three months will likely remain at one year despite some patients increasing or decreasing their ratings of satisfaction. Authors have reported that validated arthroplasty-specific patient-reported outcome measures, such as the KOOS JR, may plateau at post-operative one year and have asserted that additional follow-ups for these measures beyond this time-point are not necessary [54]. However, recent reports suggest that clinically important changes can be detected within these questionnaires up to post-operative 5 years [55], while satisfaction has been shown to improve up to post-operative 20 years [56]. Additional research to understand the trajectory of patient perception of recovery and function and the relationship to objective measures of function are needed for a better understanding of this phenomenon and a holistic view of recovery.

To the best of our knowledge, only one other study has statistically compared clinical outcomes between dissatisfied and neutral patients. In agreement with our findings, Ulivi et al. [38] reported significant differences between dissatisfied and neutral patients in KOOS-PS at post-operative five years. In contrast to our findings, there were no differences between dissatisfied and neutral patients for pain at five years nor general or physical health at either post-operative one or five years. However, the primary purpose of Ulivi et al.’s study was not to describe neutral patients, and it was likely underpowered to detect significant differences between neutral (n = 34) and dissatisfied (n = 17) patients. Two other studies seem to support a difference in clinical outcomes between neutral and dissatisfied patients. Scott et al. [23] separated improvements in Oxford Knee Score (OKS) pain and function domains at post-operative six months and presented the percentage of dissatisfied, unsure, satisfied, and very satisfied patients in the following categories: worsening; no improvement; and improvements of 1–5 points, 6–10 points, 11–15 points, and 16–20 points. A visually apparent difference was noted for OKS pain but not function. Patients that reported worsening OKS pain comprised approximately 35% dissatisfied patients compared to 20% neutral patients, with the remaining patients either satisfied or very satisfied. In contrast, no improvement in pain comprised approximately 45% neutral patients and only 15% dissatisfied patients. Dunbar et al. [12] also presented visually apparent differences in OKS 12 scores between very satisfied, satisfied, unsure, and dissatisfied patients. While no authors have reported enough information to conduct a statistical analysis, our results, combined with these studies [12,23,38], support the hypothesis that clinical differences exist between neutral, dissatisfied, and satisfied TKA patients.

The International Society of Arthroplasty Registries (ISAR) currently recommends including neutral patients as dissatisfied when assessing satisfaction [30]. However, the results of the present study contradict this recommendation. We found that neutral patients appreciate greater improvements in pain and ROM up to post-operative three months; function up to post-operative one year; and self-reported return to walking, driving, and other activities of daily living at a greater rate than dissatisfied patients. Three other studies also suggest differences in clinical outcomes between dissatisfied and neutral/unsure patients [12,23,38]. Although the characteristics of neutral satisfaction patients are poorly represented in the literature, these findings challenge the ISAR recommendation and highlight the need for more data on neutral patients.

It is commonly stated that 15–20% of TKA patients are dissatisfied with their procedure, and large cohort studies and systematic reviews support this statistic [7,8,9,10,11,12,13]. Most studies and systematic reviews have followed ISAR guidelines when reporting dissatisfaction statistics; however, the results of the present study and those obtained by Ulivi et al. [38] suggest that by classifying neutral satisfaction patients as dissatisfied, the rate of dissatisfaction following TKA has likely been overestimated. In registry and large cohort studies, classifying neutral patients as neutral would lead to a change in the reported rate of dissatisfaction from 17% [12] and 12.7% [32] to 8% and 6.8%, respectively. Further challenging the 15–20% dissatisfaction status quo, a recent meta-analysis reported a rate of dissatisfaction of 10% (ranging from 5.0–16.3%) when neutral patients were either excluded from the dissatisfied cohort or by recalculating the rate of dissatisfaction to exclude neutral patients from the dissatisfaction average [15].

The results of our study should be interpreted with caution, as we could not account for all potential confounding variables that could impact satisfaction. While generalizability is strengthened by the multi-surgeon nature of this investigation, it was not possible to control for the type of implants, surgical techniques, or post-operative protocols, which were dictated by each institution’s standard of care and could have impacted patient satisfaction and outcomes. Additionally, we could not account for a number of potential response biases that could be present, though studies of the questionnaires utilized in this report have indicated good validity and reliability. There are additional factors that could have impacted satisfaction and patient-reported outcomes, such as baseline mental health, expectations, and general personality characteristics that could not be controlled for; however, a recent study has suggested that for a multivariate analysis considering these variables, only age and BMI were significant predictors of satisfaction after TKA [50]. These variables are likely to impact satisfaction and perception of pain in all studies, as these are highly variable and subjective measures. It may be considered a limitation that we could not investigate the reasons for dissatisfaction in this study, nor could we explore the incidence of complications or adverse events following TKA, which was likely to impact satisfaction and patient-reported outcomes. Alternatively, Schoner and colleagues described a divergence of satisfied patients with clinically poor outcomes over a range of combinations to dissatisfied patients with clinically favorable outcomes. The authors found specific comorbidities associated with the occurrence of these combinations of binarily categorized satisfaction and outcomes and also suggest that additional individual characteristics will influence satisfaction [57]. Future studies that examine the contribution of measurable baseline factors and correlate additional objective measures of recovery to satisfaction after arthroplasty are needed.

## 5. Conclusions

Prior to TKA, neutral participants tended to be slightly older and more functional than dissatisfied participants. Post-operatively, neutral participants appreciate greater improvements in pain, ROM, and function and return to many activities of daily living at a higher frequency than dissatisfied patients but did not achieve similar levels as satisfied participants. The results of this study demonstrate that TKA patients who indicate neutral on satisfaction surveys present with unique clinical outcomes compared to both dissatisfied and satisfied patients and should be classified separately as neutral when reporting satisfaction results.

## Figures and Tables

**Table 1:** Pre-operative demographics between dissatisfied, neutral, and satisfied patients.

	Dissatisfied	Neutral	Satisfied	*p*-Value
Age, mean ± SD (n, range)	63 ± 9 (314, 41–84)	64 ± 9 (756, 29–92)	66 ± 9 (1500, 22–87)	<0.001
BMI, mean ± SD (n, range)	31.2 ±6.06 (314, 18.4–47.6)	30.9 ± 6.15 (755, 18.4–54.3)	31.2 ± 6.35 (1499, 15.5–61.5)	0.567
Pre-operative KSS, mean ± SD (n, range)	12.14 ± 7.26 (301, 0–40)	12.71 ± 7.17 (726, 0–36)	15.17 ± 8.26 (1432, 0–40)	<0.001
Sex, n (%)				
Female	208 (66.2)	497 (65.7)	889 (59.3)	0.003
Male	106 (33.8)	259 (34.3)	611 (40.7)	
Charnley class, n (%)				
Unilateral Joint Arthritis	184 (61.1)	474 (65.1)	922 (64.0)	0.760
Bilateral Joint Arthritis	97 (32.2)	205 (28.2)	427 (29.7)	
Unilateral or Bilateral Joint Arthritis, with other joints or conditions affecting function	20 (6.6)	49 (6.7)	91 (6.3)	
Pre-operative alignment, n (%)				
Normal	255 (98.5)	613 (99.0)	1172 (99.5)	0.220
Obvious valgus	3 (1.16)	3 (0.48)	4 (0.34)	
Obvious varus	1 (0.40)	3 (0.48)	2 (0.17)	
Pre-operative laxity, n (%)				
Lax	1 (0.4)	7 (1.1)	11 (0.9)	0.002
Normal	239 (92.6)	582 (94.5)	1134 (96.8)	
Tight	18 (7.0)	27 (4.4)	26 (2.2)	
Pre-operative pain rating, mean ± SD (n, range)	5.84 ± 2.0 (299, 1–10)	5.67 ± 1.98 (727, 0–10)	5.29 ± 2.17 (1436, 0–10)	<0.001

**Table 2:** Changes in KOOS JR scores between dissatisfied, neutral, and satisfied patients pre-operatively to post-operative one year.

	Dissatisfied	Neutral	Satisfied	*p*-Value
1 month, mean ± SD (n, range)	5.50 ± 13.56 (272, −61.58–48.08)	9.10 ± 12.77 (655, −25.80–65.99)	12.86 ± 13.52 (1297, −41.96–71.62)	<0.001
3 months, mean ± SD (n, range)	8.92 ± 14.89 (269, −29.07–63.72)	14.32 ± 12.69 (632, −23.33–65.99)	21.92 ± 14.42 (1255, −49.69–76.31)	<0.001
6 months, mean ± SD (n, range)	13.08 ± 16.40 (243, −36.71–76.04)	18.90 ± 14.14 (589, −47.49–63.98)	26.17 ± 14.85 (1164, −29.76–83.68)	<0.001
12 months, mean ± SD (n, range)	18.81 ± 18.72 (199, −54.84–68.66)	25.09 ± 15.36 (489, −47.49–79.91)	32.43 ± 15.47 (965, −18.61–100)	<0.001

**Table 3:** Frequency and duration of resumed activities in dissatisfied, neutral, and satisfied participants.

Return to Activity	Dissatisfied	Neutral	Satisfied	*p*-Value
Walk independently	263 (83.8%)	689 (91.1%)	1432 (95.5%)	<0.001
days, mean ± SD (n, range)	31.0 ± 23.3 (196, 0–98)	27.9 ± 20.4 (511, 0–111)	24.5 ± 20.0 (1129, 0–117)	<0.001
Drive independently	264 (84.1%)	691 (91.4%)	1433 (95.5%)	<0.001
days, mean ± SD (n, range)	35.0 ± 19.6 (205, 3–92)	32.2 ± 17.0 (505, 2–97)	30.7 ± 17.8 (1107, 2–117)	0.004
Return to work	118 (37.6%)	286 (37.8%)	643 (42.9%)	0.034
days, mean ± SD (n, range)	38.0 ± 24.4 (92, 1–90)	35.5 ± 21.7 (210, 1–111)	33.4 ± 23.0 (498, 1–117)	0.170
Sexual activity	114 (36.3%)	330 (43.7%)	759 (50.6%)	<0.001
days, mean ± SD (n, range)	40.7 ± 19.1 (78, 5–104)	37.9 ± 18.8 (213, 1–94)	38.7 ± 19.2 (534, 3–111)	0.530
Light housework	270 (86.0%)	702 (92.9%)	1421 (94.7%)	<0.001
Days, mean ± SD (n, range)	30.5 ± 20.4 (187, 2–103)	26.1 ± 19.0 (468, 1–96)	23.3 ± 17.3 (1023, 1–117)	<0.001
Heavy housework	71 (22.6%)	272 (36.0%)	735 (49.0%)	< 0.001
Days, mean ± SD (n, range)	45.4 ± 22.6 (61, 7–96)	45.8 ± 20.0 (209, 7–112)	45.4 ± 21.6 (556, 5–117)	0.980

## Data Availability

The data sets generated and/or analyzed during the current study are not publicly available due to proprietary information.
